# The majority of patients with long-duration type 1 diabetes are insulin microsecretors and have functioning beta cells

**DOI:** 10.1007/s00125-013-3067-x

**Published:** 2013-10-12

**Authors:** Richard A. Oram, Angus G. Jones, Rachel E. J. Besser, Bridget A. Knight, Beverley M. Shields, Richard J. Brown, Andrew T. Hattersley, Timothy J. McDonald

**Affiliations:** 1 NIHR Exeter Clinical Research Facility, University of Exeter Medical School, Barrack Road, Exeter, UK; 2 Department of Blood Sciences, Royal Devon and Exeter NHS Foundation Trust, Exeter, UK

**Keywords:** C-peptide, Insulin, Microsecretor

## Abstract

**Aims/hypothesis:**

Classically, type 1 diabetes is thought to proceed to absolute insulin deficiency. Recently developed ultrasensitive assays capable of detecting C-peptide under 5 pmol/l now allow very low levels of C-peptide to be detected in patients with long-standing type 1 diabetes. It is not known whether this low-level endogenous insulin secretion responds to physiological stimuli. We aimed to assess how commonly low-level detectable C-peptide occurs in long-duration type 1 diabetes and whether it responds to a meal stimulus.

**Methods:**

We performed a mixed-meal tolerance test in 74 volunteers with long-duration (>5 years) type 1 diabetes, i.e. with age at diagnosis 16 (9–23) years (median [interquartile range]) and diabetes duration of 30 (19–41) years. We assessed fasting and stimulated serum C-peptide levels using an electrochemiluminescence assay (detection limit 3.3 pmol/l), and also the urinary C-peptide:creatinine ratio (UCPCR).

**Results:**

Post-stimulation serum C-peptide was detectable at very low levels (>3.3 pmol/l) in 54 of 74 (73%) patients. In all patients with detectable serum C-peptide, C-peptide either increased (*n* = 43, 80%) or stayed the same (*n* = 11) in response to a meal, with no indication of levels falling (*p* < 0.0001). With increasing disease duration, absolute C-peptide levels fell although the numbers with detectable C-peptide remained high (68%, i.e. 25 of 37 patients with >30 years duration). Similar results were obtained for UCPCR.

**Conclusions/interpretation:**

Most patients with long-duration type 1 diabetes continue to secrete very low levels of endogenous insulin, which increase after meals. This is consistent with the presence of a small number of still functional beta cells and implies that beta cells are either escaping immune attack or undergoing regeneration.

**Electronic supplementary material:**

The online version of this article (doi:10.1007/s00125-013-3067-x) contains peer-reviewed but unedited supplementary material, which is available to authorised users.

## Introduction

Type 1 diabetes is defined as a disease of progressive autoimmune destruction of beta cells, leading to absolute insulin deficiency. The decline in insulin production in patients diagnosed with type 1 diabetes is variable. Most (92–97%) patients at more than 5 years from diagnosis have a stimulated serum C-peptide value <200 pmol/l [1]. Enduring endogenous insulin production has a protective effect on microvascular complications and hypoglycaemia [2].

The prevalence of detectable C-peptide in type 1 diabetic patients depends on several factors including duration of diabetes, age at diagnosis, the type and timing of sample collection (e.g. after a stimulus), and by the sensitivity of assays used [3–5]. There have been recent improvements in the sensitivity of C-peptide assays. Thus Wang et al reported that, using an ultrasensitive ELISA, C-peptide can be detected in fasting blood samples of 43% of patients with type 1 diabetes after a median disease duration of 15 years [6]. In keeping with this finding, insulin-containing beta cells have long been identified in pancreatic autopsy specimens of type 1 diabetic patients and more recently have been demonstrated to be present in up to 88% of patients with long-duration type 1 diabetes [7–10]. It is not clear whether the very low levels of C-peptide detected and the beta cells visible in autopsy specimens reflect functioning beta cells that can respond to a physiological stimulus.

The urinary C-peptide:creatinine ratio (UCPCR) is a recently described method of assessing C-peptide production that involves using a spot urine test. It is a reliable, sensitive and specific method for assessment of insulin secretion in type 1 diabetes, with test material remaining stable for 3 days at room temperature, allowing outpatients to easily be tested [11, 12]. No studies have assessed the ability of UCPCR to detect low-level insulin production.

We aimed to assess the prevalence of detectable C-peptide in patients with long-duration type 1 diabetes and determine whether low-level C-peptide rises after a meal stimulus. This was done using serum and urine samples to assess their relative sensitivity.

## Methods

### Study participants

We recruited 74 participants who had had type 1 diabetes for longer than 5 years. Participants had either been diagnosed at less than 30 years of age (*n* = 68) or when older than 30 years and with islet autoantibodies present (*n* = 6). All patients had been on insulin since diagnosis. Of the 74 participants, 38 (51%) were male. Age at diagnosis was 16 (9–23) years, median (interquartile range [IQR]), and duration of diabetes was 30 (19–41) years, with BMI of 25 (23–28) kg/m^2^, HbA_1c_ 7.9 (7.2–9.0)% (63 [55–75] mmol/mol), insulin dose 0.55 (0.44–0.69) units per kg of body weight per day and an estimated GFR of 89 (82–102) ml min^−1^ 1.73 m^−2^.

Informed consent was obtained from all participants and the study was approved by the National Research Ethics Service Committee South West (09/H0206/25).

### Mixed-meal tolerance test

Participants attended the Exeter National Institute for Health Research (NIHR) Clinical Research Facility for a standard mixed-meal tolerance test [3, 12, 13], having fasted from midnight, not taken their usual morning insulin and fully emptied their bladder upon waking. Fasting blood was taken for measurement of C-peptide, creatinine, glucose, HbA_1c_, and GAD and islet antigen 2 autoantibodies, and a second void urine sample [11] was collected for UCPCR determination. Participants were given a standard mixed meal (Ensure Plus HP; Abbott Nutrition, Columbus, OH, USA) consisting of 6 ml/kg (maximum 360 ml) water and containing per 100 ml: 15.9 g carbohydrate, 7.9 g protein, 3.3 g fat and 125 kJ energy. Blood was taken for C-peptide and glucose analysis at 90 min post-completion of the mixed meal, with urine being collected for UCPCR determination at 120 min. All participants were asked to provide a home urine sample in a boric acid container [11, 12], taken 2 h after an evening meal (and having voided the bladder before the meal). Serum and urine samples were stored at −80°C for subsequent analysis.

### C-peptide analysis

We analysed serum C-peptide with a direct electrochemiluminescence immunoassay using mouse monoclonal anti-C-peptide antibody (Roche Diagnostics, Mannheim, Germany) on an E170 analyser (Roche). The reported limit of detection is 3.3 pmol/l with a CV of 0.6% at 33 pmol/l.

We compared our Roche assay with another assay, Ultrasensitive C-peptide ELISA (Mercodia, Sylveniusgatanm, Sweden), which is a solid-phase two-site enzyme immunoassay that uses a peroxidase-TMB (3,3′,5,5′-tetramethybenzidine) label on an automated ELISA system (Dynex DSX; Launch Diagnostics, Longfield, UK). The comparison involved dual analysis of 67 samples selected for having low levels of serum C-peptide. The reported limit of detection is 1.5 pmol/l with a CV of 5.5% at 37 pmol/l.

Urinary C-peptide was analysed on the E170 analyser (Roche) as previously described [11].

### Data analysis

As C-peptide results were not normally distributed, we used non-parametric statistical tests. A Wilcoxon’s signed rank test was used to compare fasted and stimulated C-peptide levels. McNemar’s test was used to compare proportions detectable by different limits. Bland–Altman plots were used to assess the comparability of results from the Roche and Mercodia assays. Statistical analysis was performed using IBM SPSS version 20 (IBM, Armonk, NY, USA).

## Results

### C-peptide is detectable in most people with long-duration type 1 diabetes

Serum C-peptide was detectable (>3.3 pmol/l ) in 54 of 74 (73%) patients with type 1 diabetes of more than 5 years duration when measured at 90 min post meal; in the fasted state it was detectable in 49 of 74 (66%) participants.

**Fig. 1:**
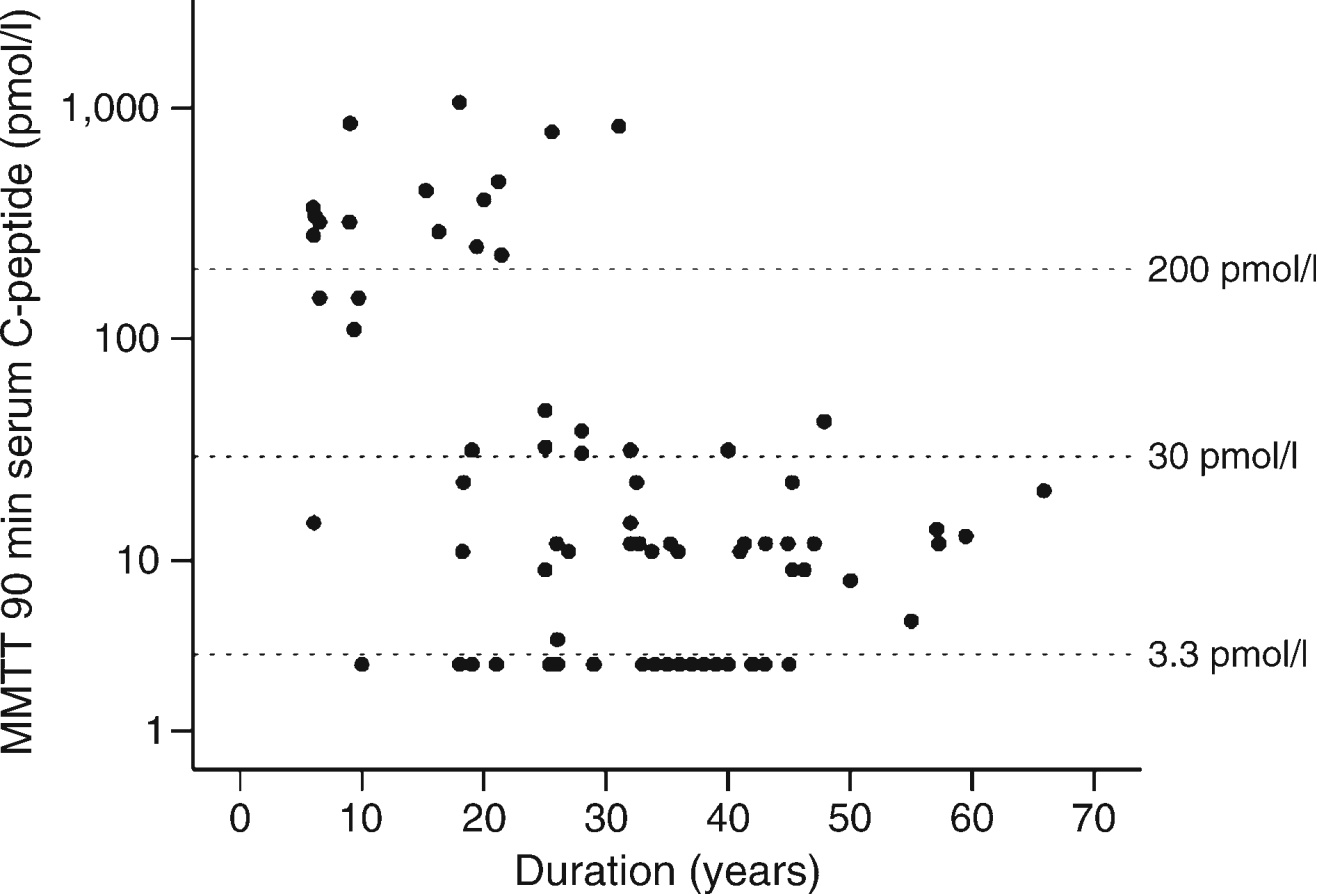
Scatterplot of serum C-peptide at 90 min after a mixed meal (log_10_ scale) against duration of diabetes. Dotted reference lines indicate 200, 30 and 3.3 pmol/l. MMTT, mixed-meal tolerance test

The detection limit makes a considerable difference to the number of patients in whom C-peptide was detected (Fig. 1). Only 35% (26 of 74, *p* < 0.0001 compared with 3.3 pmol/l) of patients had detectable stimulated C-peptide at a cut-off of 30 pmol/l, which is a previously published limit of detection [8], with 20% (15 of 74) of patients having a C-peptide value above a cut-off of 200 pmol/l, which is commonly used as a definition of significant endogenous insulin secretion [14].

The impact of using a modern assay with a lower limit of detection is more apparent with increasing disease duration. Thus in participants with diabetes of over 30 years duration, C-peptide was >3.3 pmol/l in 25 of 37 (68%) and >30 pmol/l in four of 37 (11%, *p* < 0.0001) participants.

### Low-level C-peptide is functionally responsive

**Fig. 2:**
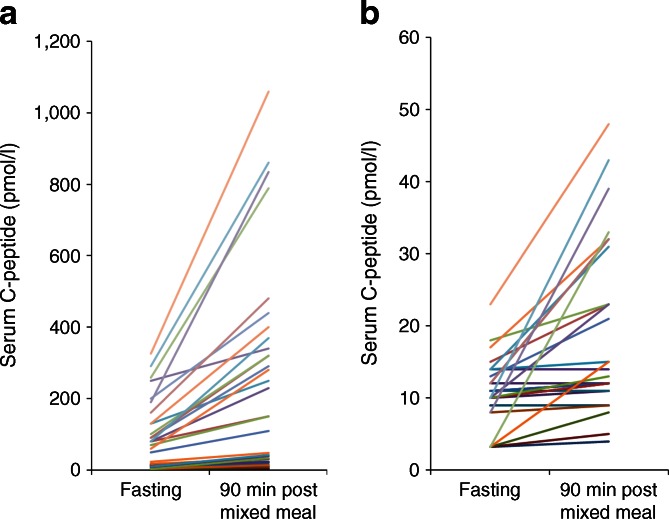
The effect of a meal stimulus on serum C-peptide levels in participants with detectable insulin (*n* = 54). (**a**) Paired fasting and mixed meal results for all patients with detectable C-peptide. Each line represents an individual patient. (**b**) Results for all patients with fasting C-peptide below 30 pmol/l (*n* = 36). Of 54 patients, 34 (80%) had a serum C-peptide value that rose after the mixed meal. None had a fall in the C-peptide value after the meal

C-peptide rose from a median (IQR) fasting value of 12 (10–80) pmol/l to 23 (12–257) pmol/l after a mixed meal (*p* < 0.0001). In all 54 patients with detectable stimulated serum C-peptide (>3.3 pmol/l), C-peptide either rose (*n* = 43, 80%) or stayed the same (*n* = 11), with none having falling levels (*p* < 0.0001) (Fig. 2). Even in 36 patients with fasting serum C-peptide <30 pmol/l, 69% (*n* = 25) had a C-peptide rise after the mixed meal.

### Low-level insulin secretion can be detected using urinary C-peptide

Low-level insulin secretion can be detected by measuring urinary C-peptide in patients with long-duration type 1 diabetes. Most patients, i.e. 51 of 74 (69%), had detectable C-peptide (>30 pmol/l) in urine taken at 120 min after the mixed meal. There was high concordance between participants who had C-peptide detected by both methods, with 50 of 51 participants with detectable urinary C-peptide also having detectable serum C-peptide. In the 51 patients with detectable urinary C-peptide, the UCPCR rose in 46 (90%) and fell in five (*p* < 0.0001) after a mixed meal. Samples taken at home 2 h after an evening meal and while on insulin therapy showed similar results (Table 1).

**Table 1:** Summary data for serum and urinary C-peptide results using the Roche assay

Variable	n	Median (IQR)	Detectable C-peptide percentage (fraction)
Fasting serum C-peptide (pmol/l)	74	11 (0–30)	73 (54/74)
Serum C-peptide at 90 min after MMTT (pmol/l)	74	12 (0–63)	66 (49/74)
Fasting UCPCR from 2nd voiding (nmol/mmol)	72	0.01 (0–0.08)	68 (49/72)
UCPCR at 120 min after MMTT (nmol/mmol)	74	0.03 (0–0.18)	68 (50/74)
UCPCR after home meal (nmol/mmol)	72	0.01 (0–0.09)	68 (49/72)

MMTT, mixed-meal tolerance test

### C-peptide measurement and duration of diabetes

Absolute C-peptide levels fell as duration of diabetes increased (Spearman’s correlation −0.46, *p* < 0.0001) (Fig. 1). However, the 20 patients with undetectable stimulated serum C-peptide had a similar disease duration to those with detectable C-peptide, i.e. 27 (18–42) vs 35 (26–40) years (median [IQR], *p* = 0.3).

### Comparison of serum C-peptide assay methods

The Roche assay was able to measure C-peptide in samples in which it was not measurable by the Mercodia assay, although the former apparently has a higher limit of detection (3.3 vs 1.5 pmol/l) (see electronic supplementary material [ESM] Results). The Mercodia assay consistently gave a lower C-peptide value for the same samples than did the Roche assay (14 vs 36 pmol/l, *p* < 0.0001, by the Bland–Altman plot) (ESM Fig. 1), showing a proportional bias with the largest difference at higher levels of C-peptide.

## Discussion

We found persistent C-peptide secretion, which increased after a meal stimulus, in the majority of patients with type 1 diabetes and a disease duration of more than 5 years. We propose that patients with a detectable stimulated C-peptide value of <30 pmol/l, which would not have been detected with older assay technologies, should be called insulin microsecretors.

Our finding that 73% of patients with type 1 diabetes of more than 5 years duration (median 29 years) had detectable C-peptide is consistent with previous studies. In a recent study, Wang et al demonstrated that 43% of patients with type 1 diabetes had detectable fasting C-peptide after a median disease duration of 15 years [6]. In Joslin medallists (diabetes duration >50 years), 67% had a detectable random C-peptide (>30 pmol/l). Indeed, it was suggested that this enduring C-peptide may have contributed to the patients’ survival [8].

An important finding is the response of very low-level C-peptide to a mixed meal. This strongly supports the notion that the residual beta cells are functional and excludes the possibility that very low levels of C-peptide are the result of analytical noise. Other studies have not directly addressed the responsiveness of these patients with very low C-peptide levels. In Joslin medallists, the response to a mixed meal was only studied in those with C-peptide over 100 pmol/l [8], but we report here that a response occurred in the majority of patients with a C-peptide concentration <30 pmol/l. Wang et al reported a correlation between glucose levels and low-level C-peptide in three patients with serial random C-peptide measurements over 3 months, but did not look at the direct response to a meal [6].

Our results provide clear evidence that the infrequent beta cells seen in histological studies of long-duration type 1 diabetes are still functional. Occasional beta cells have been noted in histological studies of patients with long-standing type 1 diabetes since 1965 [9, 10]. These were shown recently to be present in the pancreases of most patients [7, 8]. The received wisdom was that these beta cells were not functional, as older assays could not detect C-peptide in long-standing cases of type 1 diabetes. Our finding that low-level C-peptide in people with type 1 diabetes increases after a meal establishes that these rare isolated islets are functional.

We detected low-level C-peptide production not only in analyses of serum, but also of urine. This is an important point for future studies. The stability and non-invasiveness of single sample UCPCR means tests can be done at home. This could facilitate large community/cohort studies and provide a way to easily identify insulin microsecretors for future investigation.

Our study has limitations. The patients studied were not randomly selected from a population-based sample of patients with type 1 diabetes as would be the case for a true epidemiological cohort. We cannot rule out the possibility that some patients with non-type 1 diabetes were inadvertently included, although this is unlikely to be a major source of error, as a tight definition of type 1 diabetes was used and the absolute levels of C-peptide found were far lower than those described in type 2 diabetes or MODY [15]. The lack of assay standardisation makes it difficult to compare the prevalence of persistent C-peptide production when assessed by different assays [16, 17]; our comparison of the methods showed that the Roche assay detects low-level C-peptide in more patients than the Mercodia assay.

Further work is needed to establish the clinical significance and cause of insulin microsecretion. The benefits of residual insulin microsecretion in terms of hypoglycaemia, microvascular complications and long-term survival are unknown, but any impact is likely to be small so that further studies will need to have larger numbers of participants. The immunological and genetic characterisation of patients with and without residual C-peptide may help to explain why some people with type 1 diabetes have enduring endogenous insulin secretion and some do not.

In conclusion, we were able to detect enduring low-level insulin production in the majority of patients with type 1 diabetes, even in those with very long disease duration. The increase in C-peptide levels after a meal provides convincing evidence of functionally responsive beta cells. This implies that beta cells are either escaping immune attack or undergoing regeneration.

## Electronic supplementary material

Below is the link to the electronic supplementary material.

ESM Results (PDF 40 kb)

ESM Fig. 1 (PDF 54 kb)

## Abbreviations

IQR: Interquartile range
NIHR: National Institute for Health Research
UCPCR: Urinary C-peptide:creatinine ratio
