# Supplementary material for: The majority of patients with long-duration type 1 diabetes are insulin microsecretors and have functioning beta cells
Source: Diabetologia. 2013 Oct 12;57(1):187–91. doi: 10.1007/s00125-013-3067-x (PMC3855529; doi:10.1007/s00125-013-3067-x)
Supplement: Supplementary file 1 — (PDF 40 kb) [file 125_2013_3067_MOESM1_ESM.pdf]

Electronic Supplementary Material

Supplementary Results

1. Bland Altman plot for Roche v Mercodia assay is shown in supplementary figure 1.

2. Detection of low concentrations of C-peptide by the Roche and Mercodia assay.

In the 67 samples selected for having low or undetectable levels of C-peptide (C-peptide was detectable in 24/67 samples using the ROCHE assay and only 15/67 samples using the Mercodia assay, All patients detected by the Mercodia assay were also detected by the Roche assay. This suggests that the Roche assay is able to detect C-peptide at lower concentrations than the Mercodia assay.
