# Supplementary material for: The majority of patients with long-duration type 1 diabetes are insulin microsecretors and have functioning beta cells
Source: Diabetologia. 2013 Oct 12;57(1):187–91. doi: 10.1007/s00125-013-3067-x (PMC3855529; doi:10.1007/s00125-013-3067-x)
Supplement: Supplementary file 2 — (PDF 54 kb) [file 125_2013_3067_MOESM2_ESM.pdf]

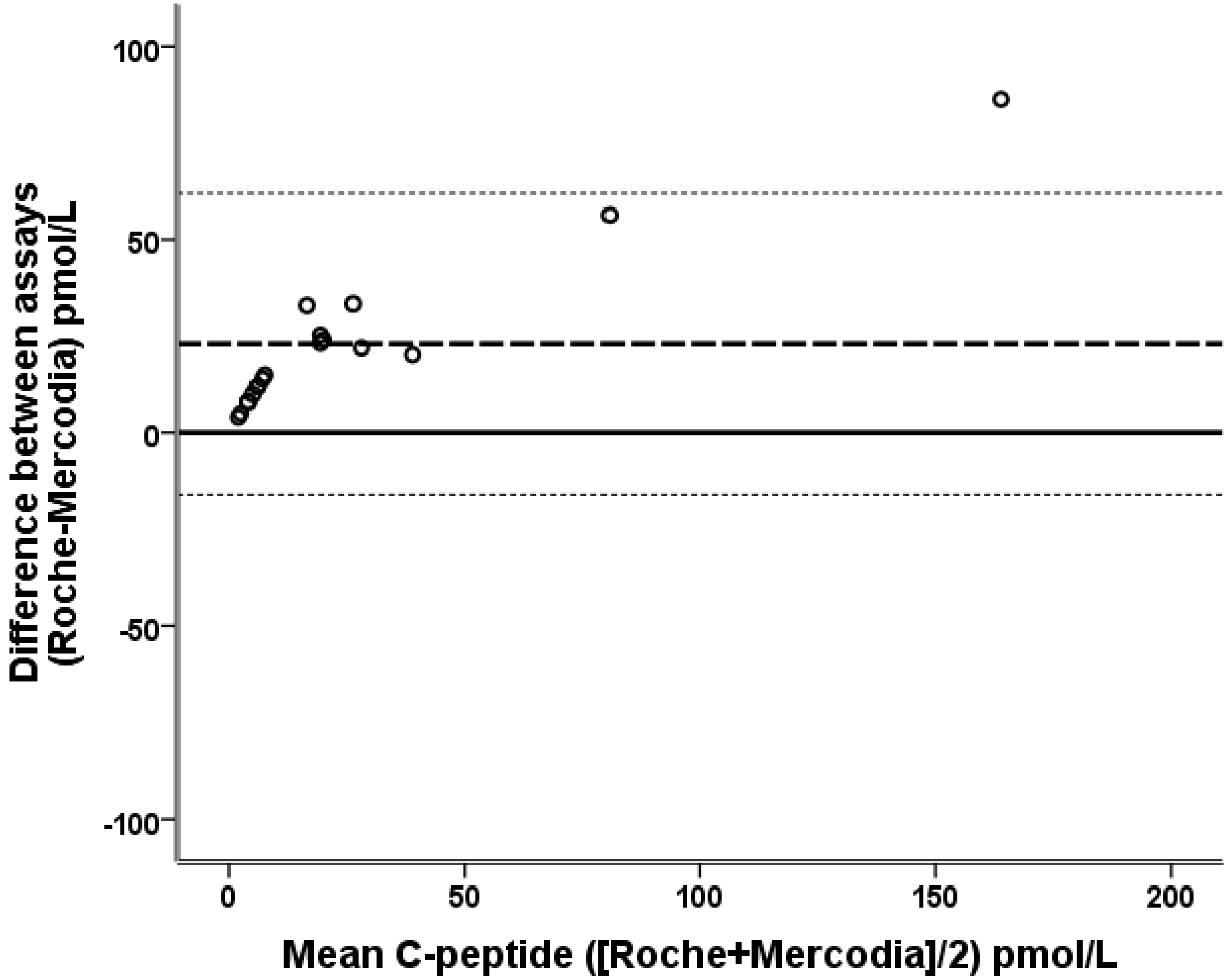

Supplementary figure 1. Bland-Altman plot comparing Roche serum C-peptide electrochemiluminescence assay with Mercodia Ultrasensitive ELISA. Mean difference (standard deviation) was 23 (20) pmol/l
